# Pharmacokinetic Monitoring of Levetiracetam in Portuguese Refractory Epileptic Patients: Effect of Gender, Weight and Concomitant Therapy

**DOI:** 10.3390/pharmaceutics12100943

**Published:** 2020-10-01

**Authors:** Rui Silva, Anabela Almeida, Joana Bicker, Joana Gonçalves, Andreia Carona, Ana Silva, Isabel Santana, Francisco Sales, Amílcar Falcão, Ana Fortuna

**Affiliations:** 1 Faculty of Pharmacy, University of Coimbra, Pólo das Ciências da Saúde, Azinhaga de Santa Comba, 3000-548 Coimbra, Portugal; rui.silva@student.ff.uc.pt (R.S.); joana.bicker@gmail.com (J.B.); joanagoncalves88@hotmail.com (J.G.); afc.carona@gmail.com (A.C.); acfalcao@ff.uc.pt (A.F.); 2 Coimbra Institute for Biomedical Imaging and Translational Research, University of Coimbra, 3000-548 Coimbra, Portugal; almeida.anabela@euvg.pt; 3 CIVG–Centro de Investigação Vasco da Gama, Escola Universitária Vasco da Gama, 3000-548 Coimbra, Portugal; 4 Refractory Epilepsy Reference Centre, Centro Hospitalar e Universitário de Coimbra, EPE, 3000-548 Coimbra, Portugal; anasilva@chuc.min-saude.pt (A.S.); isabeljsantana@gmail.com (I.S.); franciscosales@chuc.min-saude.pt (F.S.)

**Keywords:** levetiracetam, pharmacokinetic monitoring, refractory epilepsy, dosing adjustment

## Abstract

Levetiracetam is a second-generation antiepileptic drug, widely used in the treatment of focal and generalized epilepsy due to its pharmacokinetic and safety profiles. Its pharmacokinetic monitoring is ascribed as useful to personalize its dosing regimen. The aim of the present study was to describe, for the first time, the pharmacokinetics of levetiracetam in Portuguese refractory epileptic patients. Therefore, a retrospective study was carried out on 65 Portuguese refractory epileptic patients (pharmacokinetic study: 48; validation study: 17) admitted to the Refractory Epilepsy Centre of the Centro Hospitalar e Universitário de Coimbra, Coimbra, Portugal. The pharmacokinetic parameters of levetiracetam were estimated by applying a one-compartment model with first-order absorption and elimination analysis. Male patients showed higher distribution volume (Vd/F) and oral clearance (CL/F) than female patients (median Vd/F: 52.40 L in males and 38.60 L in females, *p* = 0.011; median CL/F: 4.71 L/h in males and 3.91 L/h in females, *p* = 0.028). Higher values of Vd/F (*p* = 0.026) and CL/F (*p* = 0.003) were also found in overweight patients relative to normal weight and obese patients. Carbamazepine was the co-administered antiepileptic drug that mostly affected the pharmacokinetics of levetiracetam, increasing both Vd/F (61.30 L with carbamazepine and 39.10 L without carbamazepine, *p* = 0.007) and CL/F (6.71 L/h with carbamazepine and 3.91 L/h without carbamazepine, *p* < 0.001). The pharmacokinetics of levetiracetam was affected by gender, body mass index, and co-administration of carbamazepine. This study highlights the impact of several factors on the CL/ and Vd/F of levetiracetam when administered to refractory epileptic patients. The importance of its pharmacokinetic monitoring in clinical pharmacy stands out, thereby enabling the optimization of antiepileptic drug therapy.

## 1. Introduction

Due to its efficacy and safety profiles, levetiracetam is a second-generation antiepileptic drug currently arising as the most frequently prescribed for the prophylaxis and treatment of focal and generalized epilepsy [1,2]. Although several therapeutic targets have been ascribed for levetiracetam, it is clear that it modulates the synaptic vesicle glycoprotein 2A, with consequent reduction of the release of synaptic excitatory neurotransmitters, restoring the balance between inhibitory and excitatory synaptic activities [3,4,5,6,7]. This unique mechanism of action may be linked with the usefulness of levetiracetam for the management of status epilepticus in pediatric and adult patients [8,9,10,11,12], prophylaxis of seizures from post-traumatic brain injury [13,14,15,16,17], neuropathic pain [18], and some psychiatric pathologies [19]. Moreover, its safety profile and linear pharmacokinetics, together with its low ability to cross the placental barrier, make levetiracetam a first-line option in pregnant women with epilepsy [20].

With a quick and virtually complete intestinal absorption (bioavailability > 95% and time to reach the maximum concentration of approximately 1 h), negligible plasma protein binding, distribution volume close to total body water volume (0.5 to 0.7 L/kg), and non-hepatic metabolism, levetiracetam has been considered as an antiepileptic drug with an ideal pharmacokinetic profile [21,22,23,24]. In addition, 24 h post-dosing, 90% of the administered oral dose is excreted in urine in a non-modified form (66%) and 24% as levetiracetam carboxylic acid (UCB L057). This is an inactive metabolite that results from the hydrolysis of levetiracetam, mediated by blood beta-esterases, but independent of cytochrome P450 (CYP) action [21,23]. Levetiracetam crosses the blood–brain barrier quickly and, at steady state, brain concentrations are linearly related with those found in plasma [21,23].

Regarding exposure and therapeutic response, a range of plasma concentrations between 12 and 46 mg/L (70 to 270 µmol/L) was established and applied for the prophylaxis of epileptic seizures [25,26]. However, due to the lack of correlation between drug concentrations in plasma, efficacy, and tolerability, other therapeutic ranges have been proposed [27].

Despite the aforementioned tolerability of levetiracetam and ease of dosing, monitoring its serum or plasma concentrations has been reported as useful in patients with altered physiological states, including pediatric and elderly patients [28], pregnant epileptic women [29,30,31,32], and critically ill patients [33,34,35,36,37]. Very recently, the co-administration of levetiracetam and enzyme-inducing antiepileptic drugs (EIAEDs) significantly changed the pharmacokinetics of levetiracetam [38,39]. Therefore, distinct pharmacokinetic behaviors are expected to require dosing adjustments.

Furthermore, the pharmacokinetics of levetiracetam in patients with refractory epilepsy remain unknown, as well as the factors that may modify it. A patient is considered refractory to epileptic drug treatment after the failure of adequate trials of two tolerated, appropriately chosen, and used antiepileptic drug schedules, as monotherapy or combination, to achieve sustained seizure freedom [40]. For this reason, it is of great importance to understand the pharmacokinetics of levetiracetam in refractory epileptic patients, identify intrinsic and extrinsic factors that affect its pharmacokinetics, and assess how they affect it, in order to avoid situations of apparent refractory epilepsy caused by inappropriate doses [41,42].

Thus, the present study was carried out to describe the pharmacokinetics of levetiracetam in Portuguese refractory epileptic patients. This was achieved by estimating its pharmacokinetic parameters and assessing patient characteristics that compromise them, with resort to routine pharmacokinetic data of the Refractory Epilepsy Centre of the Centro Hospitalar e Universitário de Coimbra (CHUC, EPE).

## 2. Materials and Methods

A retrospective observational study enrolled all the patients diagnosed with refractory epilepsy admitted to the Refractory Epilepsy Centre of the CHUC, EPE, Coimbra, Portugal, between January 2018 and December 2019. The patients were older than 16 years of age, under treatment with levetiracetam, and submitted to therapeutic drug monitoring as part of their routine clinical management. Patients co-administered with drugs other than antiepileptic drugs were excluded. Hence, a total of 65 Caucasian patients were herein included: 48 were part of the pharmacokinetic study, and 17 of the validation study (Table 1). Although the population should be ideally larger, it should be noted that refractory epileptic patients correspond to 1/3 of the epileptic patients, hampering the formation of study populations as big as those found for epileptic patients. The validation group consists of a completely new set of refractory epileptic patients with similar characteristics as the study population. This set allowed for the proper validation of used pharmacokinetic procedures.

Research involving human samples followed national law no. 12/2009, 26 March (Lei no 12/2009 in Diário da República no. 60/2009, Série I de 2009-03-26), which transposes to national law Directive no. 2004/23/EU of the European Parliament and Directives no. 2006/17/CE and 2006/86/CE of the European Commission. The study was approved by the Ethics Committee of CHUC (CHUC-144-18) at 3 July 2019 and by the Ethics Committee of Faculty of Medicine of University of Coimbra, Coimbra, Portugal (CE-061/2018) at 23 July 2018. All patients agreed to participate in the study and signed the informed consent. The anonymity of the sample donors (the patients) was ensured.

The following demographic, clinical, and therapeutic data were collected: gender, age (years), weight (kg), height (cm), dosing regimen of levetiracetam (dose and frequency), and all details of concomitantly administered antiepileptic drugs. Body surface area (BSA, m^2^) and body mass index (BMI, kg/m^2^) were calculated. Additionally, serum creatinine (mg/dL), serum albumin (g/dL), and blood urea nitrogen (mg/dL) were collected. Glomerular filtration rate (eGFR, mL/min/1.73 m^2^) was estimated by the Modification of Diet in Renal Disease study method, which included gender, age, serum creatinine, serum albumin, and blood urea nitrogen [43].

### 2.1. Plasma Sample Collection and Levetiracetam Quantification

Plasma sample collection was performed according to an established protocol: stationary state blood samples were collected in lithium-heparin containers approximately 30 min before levetiracetam morning administration (trough) and 1 h after (peak) (Supplementary Materials: Table S1). All drug administration and sample collection date and time were registered. When required, plasma samples were frozen at −30 °C until analysis (maximum during 48 h), guaranteeing drug stability in accordance with [44]. Quantification of levetiracetam concentrations in plasma was performed by validated liquid–liquid extraction followed by high-performance liquid chromatography (HPLC) with diode array detection (DAD) set at 220 nm. Briefly, 100 µL of plasma samples were mixed with 40 μL of methanol, 10 μL of internal standard working solution, and 1 mL of ethyl acetate. Then, the samples were centrifuged at 12,045× *g* for 3 min, in order to extract the levetiracetam dissolved in the upper organic layer. Liquid extraction with ethyl acetate was repeated and the extract was evaporated and redissolved in 100 µL of a mixture of water and acetonitrile (90/10, *v/v*), which was then analyzed by HPLC-DAD [44]. The mobile phase was composed of water and acetonitrile and pumped at 1.0 mL/min, 40 °C, using the gradient elution program described in [44].

### 2.2. Pharmacokinetic Analysis

Pharmacokinetic analysis was performed using the Abbottbase Pharmacokinetic System software (PKS^®^ System, version 1.10, Abbott Diagnostics, Green Oaks, IL, USA). A non-linear least-squares method was applied to estimate the pharmacokinetic parameters of levetiracetam using at least two drug plasma concentrations (one trough and one peak) and applying a one-compartment model with first-order absorption and elimination. This compartment model was herein chosen because it has been reported as the model that more accurately describes the pharmacokinetics of levetiracetam [38,45,46,47]. The apparent volume of distribution (Vd/F) expressed in L, oral clearance (CL/F) expressed in L/h, elimination constant rate (ke) expressed in h^−1^, and elimination half-life expressed in h, were estimated. The absorption constant rate (ka) was fixed at 2.44 h^−1^ in accordance to Rhee et al. [47]. The minimum plasma concentrations of levetiracetam at steady-state (C_MIN,SS_) were estimated for each patient according to one-compartment model equations. Additionally, the area under the plasma concentration versus time curve up to 24 h (AUC_0–24 h_), expressed in mg L/h, and the average steady-state plasma concentration (C_AV,SS_), expressed as mg/L, were calculated using the following Equations (1) and (2):

AUC_0–24 h_ (mg L/h) = [daily dose (mg)]/[CL/F (L/h)] (1)

C_AV,SS_ (mg/L) = [daily dose (mg)/24]/[CL/F (L/h)] (2)

### 2.3. Data Analysis

Patients were stratified according to their gender, BMI stage, and co-administered antiepileptic drugs. The World Health Organization classification [48] was used to stratify patients according to BMI: (1) normal weight (18.50 to 24.99 kg/m^2^), (2) overweight (25.00 to 29.99 kg/m^2^) and (3) obese (over 30.00 kg/m^2^). Pharmacokinetic parameters are presented as total Vd/F (L), total CL/F, and normalized by weight as Vd/F (L/kg) and CL/F (L/h/kg).

In order to assess the predictive performance of pharmacokinetic parameters, two error indices, the mean prediction error (ME) and the mean squared prediction error (MSE) were calculated to evaluate bias and precision, respectively, as suggested by Sheiner and Beal [49] that plays a cornerstone role in the evaluation of the predictive performance in pharmacokinetic studies. Prediction error was defined as the levetiracetam plasma concentration predicted applying the obtained Vd/F and CL/F minus the observed levetiracetam plasma concentration. The Abbottbase Pharmacokinetic System software was used to compute predicted plasma concentrations of levetiracetam of each patient. The results of ME and MSE were expressed as mean and 95% confidence interval. Root mean squared prediction error (RMSE) was calculated to measure in the units of the original quantities. The smaller the values of ME, MSE, and RMSE are, the greater the bias and precision of the models should be.

### 2.4. Statistical Analysis

All statistical analysis was carried out using the Statistical Package for the Social Sciences 26.0 (IBM SPSS^®^, Armonk, NY, USA). The results of the quantitative variables were described as mean and standard deviation when normal distribution was followed, and as median and 25th and 75th quartiles when normal distribution was not obtained. When normal distribution was followed, the Student’s *t*-test and variance analysis (ANOVA) were applied to compare the differences between two groups and more than two groups, respectively. On the other hand, for non-normal distribution, non-parametric tests were used. The Mann–Whitney U and Kruskal–Wallis H tests were used to compare the differences between two groups and more than two groups, respectively. Bonferroni correction of *p*-value was used in *post hoc* analysis. Correlations between quantitative variables were assessed by the Pearson coefficient.

## 3. Results

A total of 48 pharmacokinetic profiles from an equal number of epileptic patients (19 males and 29 females, Table 1) were included in the pharmacokinetic study. Table 2 summarizes the results of the pharmacokinetic parameters of levetiracetam obtained according to patient gender. Statistically significant differences were observed in total Vd/F (*p* = 0.011) and total CL/F (*p* = 0.028) of levetiracetam between male and female patients. However, these differences were not observed when Vd/F and CL/F were normalized by weight. Regarding weight (median values of 63.00 (52.00 to 80.50) kg and 76.00 (65.00 to 87.00) kg for women and men, respectively) or BMI (mean values of 25.42 ± 5.75 kg/m^2^ for females and 24.38 ± 4.42 kg/m^2^ for males) no statistical difference was revealed. In opposition, mean BSA observed in males (1.89 ± 0.20 m^2^) and females (1.69 ± 0.21 m^2^) were statistically different (*p* = 0.001), as well as the mean eGFR between males and females (*p* = 0.012). No statistically significant differences were observed in the elimination half-life of levetiracetam between genders.

Pearson’s correlation coefficient was used to examine the influence of weight-related parameters on pharmacokinetic parameters. However, no correlations were found between weight, BSA or BMI, and any pharmacokinetic parameters of levetiracetam. Therefore, BMI was stratified in three BMI stages, as previously described in the Material and Methods section, and the correspondent pharmacokinetic parameters of levetiracetam are summarized in Table 3. It is noteworthy the statistically significant differences were found in total Vd/F (*p* = 0.026) and total CL/F (*p* = 0.003) between normal weight (n = 26) and overweight (n = 14) groups. Significant differences were also identified between the obese group (n = 8) and normal weight (*p* = 0.019) and overweight (*p* = 0.034) groups, when Vd/F was normalized by weight. No statistically significant differences in eGFR among BMI stages were found.

No correlation was observed between eGFR and total CL/F of levetiracetam and similar results were found when CL/F was normalized by weight.

Eight patients were treated only with levetiracetam while the remaining 40 patients received, at least, one more antiepileptic drug. The results of the pharmacokinetic parameters of levetiracetam according to the concomitant antiepileptic drugs are summarized in Table 4. No statistically significant differences were found between the group of patients undergoing monotherapy (n = 8) and patients undergoing polytherapy (n = 40).

Nevertheless, statistically significant differences were observed in total Vd/F (*p* = 0.007), total CL/F (*p* < 0.001) and CL/F normalized by weight (*p* = 0.001) between the group taking carbamazepine (n = 9) and the group not taking carbamazepine (n = 39). When the group submitted to carbamazepine treatment was compared with the group undergoing monotherapy (n = 8), statistically significant differences were observed in total Vd/F (*p* = 0.014), total CL/F (*p* = 0.001), and CL/F expressed by weight (*p* = 0.010). Regarding elimination half-life, although no statistically significant differences were observed when the group taking carbamazepine was compared with either the group without carbamazepine or the group undergoing monotherapy, the group of patients taking carbamazepine showed an high half-live time. Also, statistically significant differences were observed in levetiracetam elimination half-life (*p* = 0.050) between the group taking zonisamide (n = 7) and the group not taking zonisamide (n = 41), and in the total Vd/F (*p* = 0.020) between the groups submitted (n = 12) and not submitted (n = 37) to valproic acid treatment.

**Figure 1:**
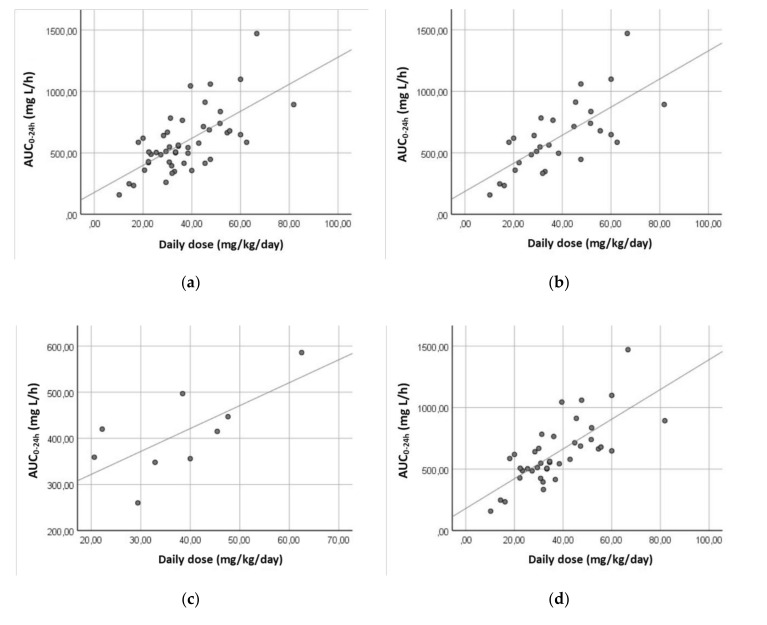
Correlation between AUC_0–24 h_ (mg L/h) and daily dose (mg/kg/day) of levetiracetam according to total sample (r^2^ = 0.433) (**a**), female patients (r^2^ = 0.505) (**b**), patients taking carbamazepine (r^2^ = 0.482) (**c**), and patients not taking carbamazepine (r^2^ = 0.527) (**d**).

AUC_0–24 h_ and C_AV,SS_ were estimated as function of CL/F and daily dose of levetiracetam, as described previously in Equations (1) and (2). The best correlation between dose, AUC_0–24 h_ and C_AV,SS_ was observed when dose was fitted by weight (r^2^ = 0.433; *p* < 0.001). This correlation improves in the female group (r^2^ = 0.505; *p* < 0.001) but worsens in the male group (r^2^ = 0.117; *p* =152). Lower correlations were observed in normal weight (r^2^ = 0.426; *p* < 0.001), overweight (r^2^ = 0.127; *p* = 0.211) and obese (r^2^ = 0.386; *p* = 0.100) patient groups. The correlation between dose, AUC_0–24 h_ and C_AV,SS_ improved when the group taking carbamazepine (r^2^ = 0.482; *p* = 0.038) and the group not taking carbamazepine (r^2^ = 0.527; *p* < 0.001) were analyzed separately (Figure 1). Statistically significant differences in AUC_0–24 h_ and C_AV,SS_ were observed when the group of patients taking carbamazepine was compared with the group not taking carbamazepine (*p* = 0.003) and the group undergoing monotherapy (*p* = 0.007) (Table 4).

Furthermore, statistically significant differences (*p* = 0.006) regarding C_MIN,SS_ were also found between patients taking and not taking carbamazepine and between patients taking and not taking zonisamide (*p* = 0.033) (Table 5). The group of patients undergoing carbamazepine presented the lowest mean value of C_MIN,SS_ (8.80 mg/L) of the studied population.

The predictive performance of the obtained pharmacokinetic parameters was evaluated by prior prediction of levetiracetam plasma concentrations in an independent set of patients (Table 1). An ME of 0.117 mg/L (−1.216; 1.034), an MSE of 5.466 mg/L^2^ (3.085; 8.337) and an RMSE of 2.338 mg/L were found, indicating a slight over-prediction of the plasma concentrations of levetiracetam.

## 4. Discussion

This study assessed the influence of gender, weight, and co-administered anti-epileptic drugs on the pharmacokinetics of orally administered levetiracetam in Portuguese refractory epileptic patients.

Male patients showed a significant increase of 36% in total Vd/F and 20% in total CL/F, relatively, to female patients (Table 2). These differences were not observed when Vd/F and CL/F were normalized by weight, suggesting that they are probably related to weight differences between the genders. Similarly, Alzueta et al. [50] reported a 14% lower CL/F of levetiracetam in females compared to males, corroborating that the underlying cause was patient weight. In this regard, the weight and BMI of male and female patients of this study were investigated, but the observed values were comparable, with no statistical differences between them. In opposition, BSA and eGFR values were statistically different between male and female patients. Thus, we suggest that the observed differences between male and female patients are a consequence of multiple physiological characteristics. Interestingly, a trend was observed with shorter half-lives in females [6.89 (5.73 to 8.12) h versus 7.63 (6.58 to 10.54) h] in males. These results corroborate those previously reported by Perucca et al. [51] who also found the same trend in the analyzed data from four clinical trials which included adult patients with refractory partial seizures administrated with levetiracetam as an adjunctive treatment. 

Moreover, a significant increment of 30% in total Vd/F was observed in overweight patients, relatively, to normal-weight patients. This demonstrates that BMI influences the pharmacokinetics of levetiracetam. A significant increment of 51% in CL/F was verified in overweight relatively to normal-weight patients. Obese patients revealed a non-significant increase of approximately 10% and a decrease of 27% in total CL/F relatively to normal weight and overweight patients, respectively. Alzueta et al. [50] did not find statistically significant differences between BMI groups in their study.

When considering concomitant antiepileptic therapy administered with levetiracetam, the present study highlights that both Vd/F and CL/F increase in patients on polytherapy. Although not statistically significant, patients on polytherapy showed a 30% higher total Vd/F and 40% higher total CL/F than those on monotherapy. Therefore, the effect of each concomitant antiepileptic drug was analyzed individually. Carbamazepine was the EIAED most frequently co-administered with levetiracetam in the study population (Table 1) and, contrary to the remaining antiepileptic drugs, it undoubtedly increased the Vd/F and CL/F of levetiracetam. For the first time, we showed that patients taking carbamazepine exhibit a total Vd/F that is 57% and 77% higher than patients not taking carbamazepine and patients on monotherapy, respectively. Regarding the total CL/F of levetiracetam, the values observed in patients taking carbamazepine increased 71% and 112%, relatively, to the aforementioned groups. Likewise, a 75% and a 91% higher CL/F per kilogram were observed in patients taking carbamazepine relatively to patients not taking carbamazepine and patients on monotherapy (Table 4).

The patient’s weight has been reported as the factor that mostly influences the Vd/F of levetiracetam in pediatric and adult Asiatic and Western non-refractory epileptic patients [38,45,47,52,53]. However, in addition to gender and weight, we also demonstrated that co-medication with carbamazepine determines the Vd/F. We suggest that hyponatremia, a well-known adverse effect of carbamazepine [54,55,56,57,58,59], may be involved in this drug–drug interaction. Briefly, the increased secretion of the antidiuretic hormone due to carbamazepine stimulation may lead to the syndrome of inappropriate antidiuretic hormone secretion. In turn, this may cause the direct stimulation of the hormone receptor and nephrogenic syndrome of inappropriate diuresis [57,60,61]. High water reabsorption appears to increase body water content with a consequent decrease of plasma sodium concentrations and decrease of blood osmolarity. Therefore, the same mechanism may be responsible for the higher median value of levetiracetam Vd/F found in patients taking carbamazepine [61.30 (43.55 to 108.60) L] relatively to patients not taking carbamazepine [39.10 (33.70 to 50.80) L] and patients on monotherapy (34.65 (28.33 to 42.80) L). Since Vd/F is a determining parameter for the establishment of drug dose, higher Vd/F may require higher doses to achieve the desired plasma concentrations.

Furthermore, carbamazepine significantly increased the total CL/F of levetiracetam (Table 4) in alignment with three previous studies [51,62,63], although the mechanisms underlying this drug interaction have not yet been clarified. Levetiracetam is mainly eliminated in parent drug form by the kidneys and to a minor extent as inactive metabolites by non-hepatic metabolism. Freitas-Lima et al. [64] hypothesized that the increase of levetiracetam CL/F might be due to an increase of non-renal clearance. However, the results of this investigation excluded the induction of hydrolytic enzymes by EIAEDs and the authors suggested a stimulation of an alternative metabolic pathway mediated by CYP isoenzymes, still unidentified, that could lead to the formation of secondary metabolites.

Although the observed values of CL/F of levetiracetam were significantly higher in patients co-administered with carbamazepine, the elimination half-life time observed in this group was shorter but not statistically significant. This suggests that patients on polytherapy with carbamazepine require higher doses but not shorter dosing intervals.

Interestingly, for the first time, we observed that the Vd/F of levetiracetam increased about 30% when co-administered with valproic acid, a classic enzymatic inhibitor, and the elimination half-life was 46% higher in patients taking zonisamide (Table 4). This is a controversial topic because Coupez et al. [65] stated that the pharmacokinetics of levetiracetam in healthy volunteers do not differ when co-administered with valproic acid, whereas Perucca et al. [51] demonstrated that valproic acid tends to decrease the CL/F of levetiracetam and consequently increase its half-life time and plasma concentrations in patients with refractory partial seizures.

Bearing the aforementioned results in mind, AUC_0–24 h_ and C_AV,SS_, were estimated in order to assess the exposure to levetiracetam. The best correlation between dose, AUC_0–24 h_, and C_AV,SS_ was observed when the dose was fitted by weight (r^2^ = 0.433) and it improves in the female group after gender stratification. Likewise, this correlation improves when patients were stratified according to the co-administration of carbamazepine (Figure 1). Significant differences were observed between the group of patients taking carbamazepine and the group not co-administered with carbamazepine or the group undergoing monotherapy (Table 4). This highlighted the importance of polytherapy with carbamazepine, besides weight and gender, on the establishment of the dosing requirements of levetiracetam. Indeed, thee higher values of Vd/F and CL/F of patients co-administered with carbamazepine lead them to present the lowest value of C_MIN,SS_ (8.80 mg/L, Table 5), which is subtherapeutic and sustains the need to increase the administered drug dose. Importantly, patients under monotherapy took lower doses of levetiracetam (median: 1750 mg/day) than patients under polytherapy (median: 3000 mg/day), however, the monotherapy group showed higher Cmin concentrations (median: 14.02 mg/L) than those of the polytherapy group (median: 12.64 mg/L) (Table 5), highlighting the importance of the effect of polytherapy on the pharmacokinetics of levetiracetam. It would be interesting to compare the monotherapy group with those administered with levetiracetam and two, three, or four AEDs but it would require a higher number of patients for each subgroup.

This is the first study reporting the influence of the concomitant administration of CYP-inducing carbamazepine on the Vd/F of levetiracetam. As formerly referred, the high Vd/F of levetiracetam found in the group taking carbamazepine is probably due to the antidiuretic effect of carbamazepine not yet studied in patients with this drug combination. Several population pharmacokinetic studies including epileptic patients were performed by nonlinear mix effects modeling and found weight as the covariate that mostly affects the Vd/F of levetiracetam [38,45,47,52,53], but none addressed the effect of carbamazepine.

Importantly, to demonstrate the accuracy of our results, the obtained pharmacokinetic parameters were validated in an independent set of patients (n = 17, Table 1). Pharmacokinetic parameters were accurate and precise, presenting an ME (0.117 mg/L) close to the theoretical value of zero and an RMSE of 2.338 mg/L. Thus, these pharmacokinetic parameters may be employed in clinical practice to estimate the dose of levetiracetam needed to achieve the desired plasma concentration, according to individual patient characteristics, as well as to perform dosage adjustments through a Bayesian approach.

## 5. Conclusions

The present investigation demonstrated that the pharmacokinetics of levetiracetam in Portuguese epileptic refractory patients is affected mainly by gender, body mass index, and co-administration of carbamazepine with consequences on drug exposure and plasma concentrations. 

To the best of our knowledge, this is the first study that assessed the pharmacokinetics of levetiracetam in a European population (namely Portuguese) and the first including only refractory epileptic patients. Moreover, complementarily to other clinical studies, we assessed the clinical impact of several covariates on the pharmacokinetics of levetiracetam, instead of only identifying them. Importantly, we demonstrated the effect of carbamazepine on levetiracetam Vd/F and not only on CL/F. This is a major clinical finding, particularly relevant to define the dose that should be administered and the need for dose adjustments in acute pathological situations (e.g., infections, surgeries, etc.) or refractory epileptic patients who are polymedicated and often not seizure-free. 

These findings highlight the importance of pharmacokinetic monitoring to personalize drug treatment with levetiracetam, in order to achieve the individual plasma concentrations necessary for seizure control without adverse effects. Population studies should be performed enrolling more patients to further elucidate the impact of each covariate in the pharmacokinetics of levetiracetam, including the impact of each AED and distinct combinations of AEDs.

## Figures and Tables

**Table 1:** Summary of the characteristics of the study population.

Characteristics	Pharmacokinetic Study	Validation Study
Patients, n	48	17
Gender, n (%)	Male: 19 (39.6%) Female: 29 (60.4%)	Male: 11 (64.7%) Female: 6 (35.3%)
Age, years	31.00 (22.25–46.50) ^b^	35.06 ± 12.36 ^a^
Weight, kg	67.00 (65.00–84.50) ^b^	77.06 ± 19.78 ^a^
Height, cm	166.65 ± 9.46 ^a^	169.41 ± 11.40 ^a^
BSA, m^2^	1.77 ± 0.23 ^a^	1.87 ± 0.28 ^a^
BMI, kg/m^2^	25.01 ± 5.24 ^a^	26.60 ± 5.28 ^a^
BMI stage		
Normal weight, n (%)	26 (54.1%)	7 (41.2%)
Overweight, n (%)	14 (29.2%)	14 (29.2%)
Obese, n (%)	8 (16.7%)	>4 (23.5%)
eGFR, mL/min/1.73 m^2^	111.57 ± 19.41 ^a^	110.97 ± 17.50 ^a^
Daily dose, mg	2750 (2000–3000) ^b^	2000 (1000–2500) ^b^
Number of daily doses
Single dosing, n (%)	1 (2.08%)	0 (0.0%)
Twice a day, n (%)	40 (88.3%)	16 (94.1%))
Three times a day, n (%)	7 (14.58%)	1 (5.9%)
Type of therapy
Monotherapy, n (%)	8 (16.7%)	7 (41.2%)
Polytherapy, n (%)	40 (83.3%)	10 (58.8%)
AEDs per patient
1 AED, n (%)	8 (16.7%)	7 (38.9%)
2 AED, n (%)	19 (39.6%)	4 (22.2%)
3 AED, n (%)	16 (33.3%)	4 (22.2%)
4 AED, n (%)	3 (6.2%)	2 (11.1%)
5 AED, n (%)	2 (4.2%)	0 (0.0%)
Concomitant antiepileptic drugs
Carbamazepine, n	9	2
Clobazam, n	6	2
Eslicarbazepine acetate, n	6	3
Lacosamide, n	2	1
Lamotrigine, n	2	0
Oxcarbazepine, n	1	0
Perampanel, n	14	5
Phenytoin, n	1	0
Topiramate, n	2	0
Valproic acid, n	12	2
Valproic acid, n	7	0

AED, antiepileptic drug; BSA, body surface area; BMI, body mass index; eGFR, estimated glomerular filtration rate. Results are expressed as ^a^ mean ± standard deviation or ^b^ median and 25th and 75th quartiles.

**Table 2:** Pharmacokinetic parameters of levetiracetam according to gender.

Pharmacokinetic Parameters	Vd/F(L)	Vd/F (L/kg)	CL/F (L/h)	CL/F (L/h/kg)	t_1/2_(h)	AUC_0–24 h_ (mg L/h)	C_AV,SS_ (mg/L)
Male (n = 19)	52.40 * (38.10–79.20)	0.749 (0.571–1.045)	4.71 * (3.64–7.23)	0.069 (0.051–0.082)	7.63 (6.58–10.54)	503.00 (415.00–579)	20.97 (17.29–24.13)
Female (n = 29)	38.60 * (33.55–47.65)	0.583 (0.514–0.738)	3.91 * (3.20–4.70)	0.058 (0.049–0.080)	6.89 (5.73–8.12)	586.00 (434.00–774.00)	24.41 (18.07–32.27)
Total (n = 48)	41.95 (34.38–54.65)	0.664 (0.532–0.799)	4.24 (3.27–5.36)	0.063 (0.051–0.082)	7.23 (5.95–8.64)	545.50 (421.25–685.00)	22.74 (17.56–28.54)

AUC_0–24 h_, area under the plasma concentration versus time curve up to 24 h; C_AV,SS_, average steady-state plasma concentration; CL/F, oral clearance; t_1/2_, elimination half-life time; Vd/F, apparent volume of distribution. Results are expressed as median and 25th and 75th quartiles. * Statistically significant difference between male and female patients (*p* < 0.05).

**Table 3:** Pharmacokinetic parameters of levetiracetam according to the BMI stage.

Pharmacokinetic Parameters	Vd/F (>L)	Vd/F (L/kg)	CL/F (L/h)	CL/F (L/h/kg)	t_1/2_ (h)	AUC_0–24 h_ (mg L/h)	C_AV,SS_ (mg/L)
Normal weight 25 < BMI (n = 26)	38.85 ^#^ (33.68–49.53)	0.696 ^$$^ (0.574–0.777)	3.62 ^##^ (2.96–4.65)	0.065 (0.051–0.085)	7.30 (6.26–8.61)	582.50 (487.30–850.30)	24.27 (20.31–35.42)
Overweight 25 ≥ BMI < 30 (n = 14)	50.30 * (43.63–120.0)	0.674 ^$^ (0.542–1.299)	5.47 ** (4.25–7.32)	0.068 (0.057–0.082)	7.81 (6.25–10.60)	499.00 (385.30–570.30)	20.79 (16.07–23.75)
Obese BMI ≥ 30 (n = 8)	37.05 (28.33–54.65)	0.486 ** ^#^ (0.334–0.565)	3.98 (3.85–5.37)	0.050 (0.041–0.058)	6.46 (4.85–7.32)	503.00 (350.80–734.00)	20.96 (14.62–3 0.60)
Total (n = 48)	41.95 (34.38–54.65)	0.664 (0.532–0.799)	4.24 (3.27–5.36)	0.063 (0.051–0.082)	7.23 (5.95–8.64)	545.50 (421.25–685.00)	22.74 (17.56–28.54)

Vd/F, apparent volume of distribution; CL/F, oral clearance; t_1/2_, elimination half-life time; AUC_0–24 h_, area under the plasma concentration versus time curve up to 24 h; C_AV,SS_, average steady-state plasma concentration. Results are expressed as median and 25th and 75th quartiles. * Statistically significant differences with normal weight group (*p* < 0.05). ** Statistically significant differences with normal weight group (*p* < 0.01). ^#^ Statistically significant differences with overweight group (*p* < 0.05). ^##^ Statistically significant differences with overweight group (*p* < 0.01). ^$^ Statistically significant differences with obese group (*p* < 0.05). ^$$^ Statistically significant differences with obese group (*p* < 0.01).

**Table 4:** Pharmacokinetic parameters of levetiracetam according to concomitant antiepileptic drugs.

Pharmacokinetic Parameters	Vd/F (L)	Vd/F (L/kg)	CL/F (L/h)	CL/F (L/h/kg)	t_1/2_ (h)	AUC_0–24 h_ (mg L/h)	C_AV,SS_ (mg/L)
Monotherapy (n = 8)	34.65 (28.33–42.80)	0.530 (0.455–0.688)	3.16 (2.76–4.38)	0.056 (0.035–0.075)	7.18 (5.91–9.45)	535.50 (349.25–949.75)	22.32 (14.56–39.58)
Polytherapy (n = 40)	45.00 (35.43–56.98)	0.684 (0.571–0.805)	4.46 (3.60–5.50)	0.065 (0.051–0.082)	7.30 (6.03–8.50)	545.50 (425.75–685.0)	22.74 (17.73–28.54)
With carbamazepine (n = 9)	61.30 ** ^#^ (43.55–108.60)	0.807 (0.577–1.783)	6.71 *** ^###^ (5.35–7.83)	0.107 *** ^#^ (0.067–0.111)	6.39 (4.86–11.19)	415.00 ** ^##^ (352.00–472.00)	17.29 ** ^##^ (14.68–19.67)
Without carbamazepine (n = 39)	39.10 (33.70–50.80)	0.652 (0.503–0.751)	3.91 (3.09–4.63)	0.061 (0.047–0.071)	7.58 (6.37–8.60)	579.00 (488.00–740.00)	24.13 (20.33–30.82)
With clobazam (n = 6)	47.20 (38.65–55.95)	0.748 (0.565–0.934)	4.53 (3.66–5.69)	0.078 (0.045–0.097)	6.96 (6.19–8.51)	663.50 (538.00–835.00)	27.64 (22.42–34.81)
Without clobazam (n = 42)	40.70 (33.68–55.93)	0.656 (0.521–0.796)	4.15 (3.21–5.45)	0.062 (0.051–0.078)	7.52 (5.91–8.69)	509.50 (418.75–672.75)	21.21 (14.46–28.04)
With eslicarbazepine acetate (n = 6)	36.45 (28.88–53.33)	0.564 (0.389–0.759)	4.60 (3.17–5.95)	0.066 (0.045–0.076)	6.69 (5.37–7.71)	553.00 (422.50–868.75)	23.06 (17.59–36.20)
Without eslicarbazepine acetate (n = 42)	43.80 (34.53–55.93)	0.674 (0.557–0.802)	4.15 (3.21–5.24)	0.063 (0.051–0.082)	7.52 (5.60–8.99)	530.00 (418.75–693.75)	22.07 (17.46–28.91)
With perampanel (n = 14)	42.25 (37.53–54.30)	0.689 (0.569–0.827)	4.01 (3.48–5.14)	0.063 (0.048–0.074)	7.90 (6.39–9.65)	616.50 (502.25–769.25)	25.67 (20.92–33-19)
Without perampanel (n = 34)	41.95 (33.68–55.93)	0.656 (0.502–0.797)	4.49 (3.20–5.68)	0.064 (0.051–0.084)	6.98 (5.88–8.20)	505.00 (410.00–652.00)	21.04 (17.09–27.16)
With zonisamide (n = 7)	50.80 (41.60–60.00)	0.698 (0.660–1.045)	4.10 (2.87–5.12)	0.070 (0.048–0.107)	10.14 * (6.89–12.08)	664.00 (488.00–912.00)	27.65 (20.33–38.02)
Without zonisamide (n = 41)	39.20 (34.00–52.40)	0.600 (0.502–0.782)	4.27 (3.39–5.47)	0.062 (0.052–0.079)	6.95 (5.90–8.08)	512.00 (415.00–673.50)	21.31 (17.29–28.06)
With valproic acid (n = 12)	51.10 * (41.03–59.45)	0.678 (0.572–1.015)	4.60 (3.92–6.03)	0.068 (0.051–0.081)	7.61 (6.42–10.28)	582.50 (425.75–707.25)	24.26 (17.73–29.48)
Without valproic acid (n = 36)	38.80 (33.53–51.60)	0.656 (0.501–0.788)	3.98 (3.11–5.17)	0.062 (0.049–0.082)	6.96 (5.88–8.17)	545.50 (416.25–671.25)	22.74 (17.35–27.96)

Vd/F, apparent volume of distribution; CL/F, oral clearance; t_1/2_, elimination half-life time; AUC_0–24 h_, area under the plasma concentration versus time curve up to 24 h; C_AV,SS_, average steady-state plasma concentration. Results are expressed as median and 25th and 75th quartiles. * Statistically significant difference between patients taking and not taking the concomitant antiepileptic drug (*p* < 0.05). ** Statistically significant difference between patients taking and not taking the concomitant antiepileptic drug (*p* < 0.01). *** Statistically significant difference between patients taking and not taking the concomitant antiepileptic drug (*p* < 0.001). ^#^ Statistically significant differences between patients taking the concomitant antiepileptic drug and patients on monotherapy (*p* < 0.05). ^##^ Statistically significant differences between patients taking the concomitant antiepileptic drug and patients on monotherapy (*p* < 0.01). ^###^ Statistically significant differences between patients taking the concomitant antiepileptic drug and patients on monotherapy (*p* < 0.001).

**Table 5:** Daily dose and minimum plasma concentration of levetiracetam according to concomitant antiepileptic drugs.

Pharmacokinetic Parameters	Levetiracetam Daily Dose (mg/day)	C_MIN,SS_ (mg/L)
Monotherapy (n = 8)	1750 (1500–3000)	14.02 (7.27–20.62)
Polytherapy (n = 40)	3000 (2000–3000)	12.64 (9.30–17.05)
With carbamazepine (n = 9)	3000 (2250–3000)	8.80 ** (7.32–9.90)
Without carbamazepine (n = 39)	2500 (2000–3000)	13.49 (10.88–17.94)
With clobazam (n = 6)	3000 * (3000–3000)	16.47 (12.04–23.72)
Without clobazam (n = 42)	2500 (2000–3000)	12.42 (9.13–17.12)
With eslicarbazepine acetate (n = 6)	3000 (2000–3000)	11.19 (8.62–19.03)
Without eslicarbazepine acetate (n = 42)	2500 (2000–3000)	12.93 (9.43–17.24)
With perampanel (n = 14)	3000 (2000–3000)	15.72 (11.61–21.83)
Without perampanel (n = 34)	2500 (2000–3000)	11.45 (9.13–16.67)
With zonisamide (n = 7)	3000 (2500–3000)	17.19 * (13.50–27.64)
Without zonisamide (n = 41)	2500 (2000–3000)	12.35 (8.85–16.72)
With valproic acid (n = 12)	3000 (2500–3000)	12.29 (9.41–16.98)
Without valproic acid (n = 36)	2500 (1625–3000)	12.64 (9.00–17.30)

C_MIN,SS_, steady-state minimum plasma concentration. Results are expressed as median and 25th and 75th quartiles. * Statistically significant difference between patients taking and not taking the concomitant antiepileptic drug (*p* < 0.05). ** Statistically significant difference between patients taking and not taking the concomitant antiepileptic drug (*p* < 0.01).

## Supplementary Materials

The following are available online at https://www.mdpi.com/1999-4923/12/10/943/s1, Table S1: Number of patients and of levetiracetam plasma concentrations included in the analysis.
