# Supplementary material for: Pharmacokinetic Monitoring of Levetiracetam in Portuguese Refractory Epileptic Patients: Effect of Gender, Weight and Concomitant Therapy
Source: Pharmaceutics. 2020 Oct 1;12(10):943. doi: 10.3390/pharmaceutics12100943 (PMC7601255; doi:10.3390/pharmaceutics12100943)
Supplement: Supplementary file 1 [file pharmaceutics-12-00943-s001.pdf]

pharmaceutics

Supplementary Materials: Pharmacokinetic Monitoring of Levetiracetam in Portuguese Refractory Epileptic Patients: Effect of Gender, Weight and Concomitant Therapy

Rui Silva, Anabela Almeida, Joana Bicker, Joana Gonçalves, Andreia Carona, Ana Silva, Isabel Santana, Francisco Sales, Amílcar Falcão and Ana Fortuna

Table S1. Number of patients and of levetiracetam plasma concentrations included in the analysis.

	**Patients (Plasma concentrations)**	**Patients (Cmin plasma concentrations)**	**Patients (1 h post dose plasma concentrations)**	**Patients (Additional Cmin plasma concentrations)**
Patients with 2 plasma concentrations	25 (50)	25 (25)	25 (25)	0 (0)
Patients with 3 plasma concentrations	18 (54)	18 (18)	18 (18)	18 (18)
Patients with 4 plasma concentrations	5 (20)	5 (5)	5 (5)	5 (10)
**Total**	**48 (124)**	**48 (48)**	**48 (48)**	**23 (28)**

Cmin, Minimum plasma concentration
